# Health-related quality of life and lifetime QALY loss among Indigenous Australians with chronic conditions: an age-stratified analysis

**DOI:** 10.1007/s11136-026-04301-0

**Published:** 2026-06-06

**Authors:** Rezwanul Haque, Annika Luebbe, Matthew McGrail, Bushra Farah Nasir, Khorshed Alam, Katharine Wallis, Floyd Leedie, Srinivas Kondalsamy-Chennakesavan

**Affiliations:** 1 Toowoomba Regional Clinical Unit, Medical School, Faculty of Health, Medicine and Behavioural Sciences, The University of Queensland, Toowoomba, QLD 4350 Australia; 2 Rockhampton Regional Clinical Unit, Medical School, Faculty of Health, Medicine and Behavioural Sciences, The University of Queensland, Rockhampton, QLD 4700 Australia; 3 School of Business, Law, Humanities and Pathways and Centre for Health Research, University of Southern Queensland, Toowoomba, QLD 4350 Australia; 4 General Practice Clinical Unit, Medical School, The University of Queensland, Brisbane, QLD 4006 Australia; 5 Goondir Health Services, Dalby, QLD 4405 Australia

**Keywords:** Indigenous health, Health-related quality of life, Chronic disease, EQ-5D-5L, Health utility, Quality adjusted life years (QALY)

## Abstract

**Background:**

Indigenous Australians in rural and remote areas experience substantial health-related quality of life (HRQoL) impacts alongside persistent healthcare access barriers. Community-led virtual primary care services offer an innovative approach to improving access to health care services for Indigenous Australians in rural and remote areas.

**Objective:**

To examine age-stratified HRQoL patterns and estimate the lifetime quality-adjusted life year (QALY) loss among Indigenous Australians with chronic conditions enrolled in a rural virtual primary care service.

**Methods:**

We conducted a cross-sectional analysis of 75 Indigenous adults residing in rural Queensland. HRQoL was measured using the EQ-5D-5L instrument. Lifetime QALY loss was calculated using Queensland Indigenous life tables and population norms, with sensitivity analyses using Australian norms and varying discount rates.

**Results:**

Overall mean utility was 0.775 (SD = 0.246). Age-stratified analysis revealed significant heterogeneity, with three age groups (18–54, 55–64, 65–74 years) demonstrating lower HRQoL than Queensland norms. The 55–64 age group experienced poorest HRQoL (utility = 0.701, SD = 0.287) and highest projected lifetime QALY loss (4.44 QALYs undiscounted; 2.63 with 5% discount). In contrast, participants aged 75 years and above exceeded population norms (utility = 0.872 vs. 0.863). Chronic disease burden was associated with HRQoL decline in adults aged 18–64 years, while physical activity was associated with higher HRQoL in those aged 65 years and over.

**Conclusions:**

Indigenous Australians aged 55–64 years represent a critical priority for virtual primary care interventions. Targeted support strategies for this ‘at-risk’ age group are essential to address substantial lifetime health burdens and improve long-term outcomes within remote delivery models.

## Introduction

In rural and remote Australia, people with chronic diseases experience a reduced quality of life and a disproportionate burden of disease compared to their metropolitan counterparts [1]. Aboriginal and Torres Strait Islander peoples (hereafter respectfully referred to as Indigenous Australians) face substantially higher rates of chronic disease, with onset typically occurring at younger ages than in the non-Indigenous population [2]. Understanding how this burden affects health-related quality of life (HRQoL) across different age groups and identifying factors that may protect against quality of life decline is essential for designing age-appropriate interventions and allocating resources to mitigate health disparities.

HRQoL is a multidimensional construct that contextualises how physical and psychological health influences an individual’s ability to live a functional, fulfilling life through perceived wellbeing and social participation [3]. HRQoL is measured using self-reported health questionnaires that provide insight into key influential factors such as how chronic illness contributes to disease burden and determines broader wellbeing [4]. The EQ-5D-5L is one such measurement tool, developed by EuroQol and updated in 2010 to include health dimensions: mobility, self-care, usual activities, pain/discomfort, and anxiety/depression [5]. The EQ-5D-5L is regarded as a reliable and valid instrument for assessing health status, applicable to a broad range of populations and settings [6]. It has also specifically been validated as appropriate for Indigenous Australian populations to assess health status and inform economic evaluations [7].

Despite the importance of HRQoL measurement for healthcare planning and resource allocation, limited research has examined age-specific patterns and determinants among Indigenous Australians with chronic conditions. Existing evidence demonstrates that multimorbidity reduces HRQoL in Indigenous Australians, with mean utility values of 0.63 for those with multiple conditions compared to 0.77 for those with none [8]. However, critical knowledge gaps remain regarding how these HRQoL patterns vary across age groups, the projected lifetime burden when translated into Quality-Adjusted Life Year (QALY) losses using Indigenous-specific life expectancy, and whether different factors, such as chronic disease burden and modifiable lifestyle factors impact HRQoL at different life stages. Addressing these gaps is critical given that chronic disease onset occurs earlier in Indigenous populations, potentially resulting in prolonged periods of compromised quality of life during their most economically and socially productive years. However, little is known about how these patterns evolve across the lifespan or what factors may be protective in older age.

Virtual health services incorporating remote monitoring offer a promising mechanism to address geographical access barriers while supporting chronic disease management [9]. Systematic reviews demonstrate that telehealth improves access, clinical outcomes, and social and emotional wellbeing for Indigenous Australians, with high satisfaction reported by patients and families [10]. Remote monitoring extends these benefits by enabling proactive, home-based health tracking, potentially reducing emergency presentations and supporting disease management between clinical encounters [11–13]. However, implementation of this technology-enabled care requires co-design with Indigenous communities, including Indigenous governance, trained local community health workers, and integration of cultural protocols to ensure cultural safety [14].

Goondir health services is one such Aboriginal Community Controlled Health Organisation (ACCHO) that established a Virtual Health Service (VHS) program to provide chronic disease patients remote monitoring of their health. Implemented in response to growing demand during COVID-19, Goondir’s VHS sits alongside their normal services delivered to Indigenous people at four regional and remote sites across the Darling Downs and South-West Hospital and Health Service (HHS) areas. Patients received a tablet with the VHS app, alongside health monitoring devices to measure blood glucose, weight, blood pressure, and pulse [9]. Regular clinical readings are uploaded by patients using the app, while these readings are monitored remotely at Goondir clinics by trained Aboriginal Health Workers (AHWs) using a streamlined and integrated VHS dashboard. Any abnormal readings are flagged and follow-up contact made with the patient; if required a virtual (or in-person) appointment is made with the patients General Practitioner (GP). This remote monitoring approach can reduce the need for doctor appointments and has demonstrated positive patient outcomes including reduced emergency department presentations [9].

The VHS program provides a comprehensive, culturally responsive prevention and management approach for patients with chronic diseases. It has the potential to improve quality of life and reduce chronic disease burden by reducing barriers to healthcare, supporting ongoing management and strengthening feelings of safety, connection and autonomy in health decisions over time. This cross-sectional study examines baseline age-stratified HRQoL patterns among Indigenous Australians with chronic conditions enrolled in the Goondir VHS. The specific objectives are: (1) to measure HRQoL utility scores and compare them with Queensland and Australian population norms across age groups; (2) to quantify projected lifetime QALY losses using Indigenous-specific life expectancy data; and (3) to identify age-specific determinants of HRQoL, including chronic disease burden and modifiable lifestyle factors. By identifying the life stages where the health burden is most acute, these findings will inform targeted intervention strategies within virtual care models to address the specific needs of rural Indigenous Australians with chronic conditions.

## Methods

### Study design and setting

This cross-sectional study was conducted within the Innovative Digital–IndigeNouS PrImaRy hEalthcare Delivery (ID-INSPIRED) collaborative project, funded by the Australian Government through the Medical Research Futures Fund (Grant APP2023585). Data were collected between June 2024 and July 2025 from consenting enrolled VHS participants. Survey instruments were co-designed by the project Governance Committee comprising Indigenous consumers, community representatives, health leaders, and researchers. This governance structure ensured cultural appropriateness and alignment with community priorities throughout the research process. The VHS operational model, Indigenous governance framework, and service delivery context have been comprehensively described previously [9].

### Sampling frame and participants

The sampling frame comprised of all VHS users within Goondir Health Services at the time when the survey was conducted. This group comprised 153 Indigenous adults (as of June 2024) with chronic conditions residing in rural and remote regions designated Modified Monash Model (MMM) [15] 4–7, i.e. those from medium and small rural towns, and remote and very remote communities. Inclusion criteria specified Indigenous adults aged 18 years or older with at least one diagnosed chronic condition, registered as VHS users, and provided verbal or written informed consent. Data were only available from participants who provided informed consent and completed the survey. Per ethics approval and Indigenous data governance protocols, we were not permitted to access health service records for non-consenting individuals, precluding direct comparison of responders and non-responders on demographic or clinical characteristics to judge potential non-response bias.

### Ethical approval

Ethical approval for this study was obtained from the University of Queensland Human Research Ethics Committee (approval number 2022/HE002522). Informed consent was obtained from all participants following detailed explanation of the study objectives, procedures, and participant rights. Data governance was overseen by the project Governance Committee, which ensured compliance with cultural and clinical protocols and provided final approval prior to dissemination of outcomes. The study was conducted in alignment with established Indigenous research ethics and governance frameworks, including the CARE Principles for Indigenous Data Governance [16] and the NHMRC guidelines for Indigenous health research [17, 18]. These frameworks safeguarded Indigenous communities’ authority, control, and benefit in relation to research data and outcomes.

### Outcome variable

HRQoL was assessed using the EQ-5D-5L, the most widely adopted instrument globally for health economic evaluations [19, 20]. The EQ-5D-5L is particularly suited for chronic disease populations due to its brevity, well-established psychometric properties across diverse clinical and general populations, and international uptake facilitating cross-national comparisons [6, 21]. The instrument comprises five single-item dimensions: mobility, self-care, usual activities, pain/discomfort, and anxiety/depression [5]. Each dimension is rated on a 5-level severity scale: no problems (level 1), slight problems, moderate problems, severe problems, and extreme problems/unable to perform (level 5). Health utility scores were derived from the five-dimensional health state profiles using the Australian EQ-5D-5L value set [22], which reflects societal preferences elicited from a representative sample of the Australian general population. Under the Australian scoring algorithm, possible utility values of EQ-5D-5L range from − 0.30 (the worst possible health state with extreme problems across all five dimensions, valued worse than death) to 1 (perfect health), with 0 representing death [23]. The EQ-5D-5L also incorporates a Visual Analogue Scale (VAS), where respondents rate their overall health on a vertical scale anchored at 0 (worst imaginable health) to 100 (best imaginable health). While VAS scores were collected and reported descriptively, health utility values served as the primary outcome for QALY loss calculations, consistent with standard health economic methodology [24, 25].

### Independent variables

Sociodemographic variables included age (18–54, 55–64, 65–74, 75 years and above), gender (male, female), relationship status (married/partnered, separated/divorced/widowed, never married), educational attainment (secondary or below, post-secondary), employment status (employed, retired, unemployed, not in labour force, student), and individual income (low/no income, moderate income). Geographic rurality was classified using the MMM. Due to small numbers in MMM 6 and 7 categories, rurality was dichotomised as: MMM 4 (medium rural) versus MMM 5–7 (small rural, remote, and very remote communities combined). Lifestyle factors included smoking status (never smoker, ex-smoker, current smoker). Alcohol consumption was classified as former/never drinker or currently drinking. Physical activity levels were categorised according to the World Health Organisation (WHO) recommendations for adults, which suggest engaging in at least 150 min of moderate to intensity physical activity per week [26]. Chronic disease burden was the total count of self-reported chronic conditions, examined continuously and categorically (single, 2–3, 4 or more conditions).

### Statistical analysis

This study used descriptive analysis to characterise participants’ sociodemographic, geographic, lifestyle factors, chronic disease burden, and EQ-5D-5L profiles. Continuous variables are presented as mean (standard deviation) and categorical variables as frequencies and percentages. Multivariable linear regression identified independent predictors of EQ-5D-5L utility scores. Given the smaller sample size, several categorical variables were collapsed to binary form, and a parsimonious model was developed, including only theoretically important predictors, consistent with recommended regression practices for smaller sample sizes [27, 28]. Age was dichotomised as < 65 vs. ≥ 65 years to ensure adequate statistical power while maintaining consistency with the descriptive age stratification used in subsequent analyses. In the age stratified analysis, participants were classified into four age groups: 18–54, 55–64, 65–74, and 75 years and above. The broader 18–54 band was necessary as further stratification would yield subgroups below the *n* ≥ 10 threshold [27, 28]. Differences across age groups were examined using ANOVA for continuous variables and chi-square tests for categorical variables.

Participant utility scores were compared with general Australian population norms using one-sample t-tests. Primary comparisons used Queensland-specific norms [29] as the geographically matched reference, while sensitivity analyses employed Australian national norms [30]. Both norm sets derive from general (predominantly non-Indigenous) populations using the EQ-5D-5L Australian value set [22]. Utility decrements were calculated for each subgroup, providing the foundation for QALY loss estimation. Lifetime QALY loss quantified cumulative health burden over remaining lifespans through three steps. First, individual utility difference was calculated as Queensland age-specific population norm minus participant’s utility score. Positive values indicate utility below population norms (QALY loss); negative values indicate utility above norms (QALY gain). Second, individual remaining life expectancy was determined using Queensland Aboriginal and Torres Strait Islander life Tables (2020–2022) [31]. The assignment approach varied by age group alignment with ABS life table categories. For the 18–54 age group, we used midpoint approximation (age 35–39), a standard demographic method for broad age ranges [32, 33]. For the 55–64 and 65–74 groups, which each span two ABS categories, we averaged male and female life expectancy values separately across the relevant categories (55–59 and 60–64; 65–69 and 70–74 respectively), then applied gender weighting based on observed proportions within each stratum. For the 75 years and above age group, the 75–79 ABS category was used as the reference. Gender-specific life expectancy was applied throughout, with weighted averages proportional to gender distribution within each age stratum, consistent with Australian Institute of Health and Welfare burden of disease methodology [34]. Third, individual QALY loss was calculated by multiplying the utility difference by the remaining life expectancy, preserving directional information (positive = loss, negative = gain) and assuming a constant utility difference over remaining lifespan. Lifetime QALY loss was calculated using undiscounted estimates (primary analysis) and discounted estimates at 3% and 5% rates (sensitivity analyses). Undiscounted estimates may better align with Indigenous perspectives on intergenerational wellbeing by assigning equal value to quality of life across all future years, while discounting reflects time preference and uncertainty [35], with 5% aligning with Australian health economic guidelines [36]. For discounted estimates: Discounted QALY Loss = Utility Decrement × [(1 − (1/(1 + r)^T)) / r], where r = discount rate and T = remaining life years.

All analyses were conducted using Stata version 18.0 (StataCorp LLC, College Station, TX, USA). Statistical significance was set at α = 0.05 (two-tailed).

## Results

Of 153 eligible VHS participants, 75 (49.0%) consented and completed the EQ-5D-5 L assessment. This sample comprised 23 (30.7%) participants aged 18–54 years, 23 (30.7%) aged 55–64 years, 21 (28.0%) aged 65–74 years, and 8 (10.6%) aged 75 years and above.

Table 1 shows participant sociodemographic and lifestyle characteristics. Participants had a mean age of 58.4 years (SD = 14.4) and were predominantly female (76.0%). The majority had attained secondary education or below (81.3%), resided in more remote areas (MMM 5,6,7: 64.0%), and reported low or no annual income (77.3%). Employment status varied, with 40.0% retired, 28.0% employed, 14.7% unemployed, and 16.0% not in the labour force. Regarding lifestyle factors, 41.3% were never smokers, 36.0% were ex-smokers, and 22.7% current smokers, while 62.7% were former alcohol drinkers or had never drunk. Participants experienced substantial chronic disease burden, with a mean of 3.5 conditions (SD = 1.7, range 1–8). Most had multimorbidity: 50.7% reported 2–3 conditions and 41.3% had high multimorbidity (4 or more conditions). Despite this burden, 74.7% self-reported meeting recommended physical activity levels.

**Table 1:** Sociodemographic and lifestyle characteristics of study participants

Characteristic	n	Mean (SD)/(%)
**Sociodemographic factors**		
Age (years)	75	58.4 (14.41)
**Age groups**		
18–54 years	23	30.7
55–64 years	23	30.7
65–74 years	21	28.0
75 years and above	8	10.6
**Gender**		
Male	18	24.0
Female	57	76.0
**Education level**		
Secondary or below	61	81.3
Post-secondary	14	18.7
**Relationship status**		
Married/partnered	27	36.0
Separated/ divorced/widowed	26	34.7
Never married	22	29.3
**Geographic rurality**		
MMM* 4	27	36.0
MMM 5,6,7	48	64.0
**Employment status**		
Employed	21	28.0
Retired	30	40.0
Unemployed	11	14.7
Not in the labour force	12	16.0
Student	1	1.3
**Individual income (Yearly)**		
Low/No income	58	77.3
Moderate income	17	22.7
**Lifestyle factors**		
**Smoking status**		
Never smoker	31	41.3
Ex-smoker	27	36.0
Current smoker	17	22.7
**Alcohol consumption**		
Former/never drunk	47	62.7
Currently drinking	28	37.3
**Physical activity level**		
No physical activity	19	25.3
Recommended physical activity	56	74.7
**Chronic diseases burden**		
Total chronic conditions, mean (SD)	75	3.52 (1.70)
Condition range	75	1–8
**Chronic disease categories**		
Single condition	6	8.0
Multiple conditions (2–3)	38	50.7
High multimorbidity (4+)	31	41.3

*Modified Monash Model (MMM): MMM 4 = medium rural towns; MMM 5 = small rural towns; MMM 6 = remote; MMM 7 = very remote. 2. SD: Standard deviation

Table 2 summarises the EQ-5D-5L HRQoL profile of the study participants. Pain/discomfort showed the highest mean severity level (2.52, SD = 1.16), with 46.6% reporting moderate to extreme problems doing usual activities, followed by anxiety or depression (mean 1.99, SD = 1.08). Mobility, doing usual activities, and self-care showed progressively less impairment (mean scores 1.84 (SD = 0.93), 1.80 (SD = 1.07) and 1.28 (SD = 0.64) respectively). The overall mean utility score was 0.775 (SD = 0.246). Self-rated overall health on the VAS showed a mean of 65.1 (SD = 22.8) and median of 70.0 (IQR: 50.0–80.0).

**Table 2:** EQ-5D-5L health -related quality of life profile (*N* = 75)

EQ-5D-5L component	Value				
Dimension-level severity	Mean level (SD)				
Mobility	1.84 (0.93)				
Self-care	1.28 (0.64)				
Usual activities	1.80 (1.07)				
Pain/discomfort	2.52 (1.16)				
Anxiety/depression	1.99 (1.08)				

EQ-5D-5L Dimensions-Detailed distribution	No problem (level 1), n (%)	Slight problem (level 2), n (%)	Moderate problem (level 3), n (%)	Severe problem (level 4), n (%)	Extreme/unable (level 5), n (%)
Mobility	36 (48.0)	18 (24.0)	18 (24.0)	3 (4.0)	0 (0.0)
Self-care	61 (81.3)	9 (12.0)	3 (4.0)	2 (2.67)	0 (0.0)
Usual activities	42 (56.0)	14 (48.7)	12 (16.0)	6 (8.0)	1 (1.33)
Pain/discomfort	16 (21.3)	24 (32.0)	19 (25.3)	12 (16.0)	4 (5.3)
Anxiety/depression	32 (42.7)	22 (29.3)	13 (17.3)	6 (8.0)	2 (2.67)

EQ-5D-5L Summary Measures					
Utility score, Mean (SD)	0.775 (0.246)				
Utility score range	− 0.066 to 1.000				
Visual analogue scale (VAS), mean (SD)	65.1 (22.8)				
VAS, [Median (IQR)]	70 (50–80)				
VAS range	5 to 100				

(1) EQ-5D-5L utility scores calculated using Australian value set [22]; (2) SD = Standard deviation; (3) EQ-5D-5L dimensions scored from 1 (no problems) to 5 (extreme problems/unable to perform); (4) Negative utility values indicate health states considered worse than death

Table 3 presents multivariable linear regression results examining independent predictors of HRQoL. Three variables demonstrated statistically significant associations after adjusting for potential confounders. Chronic disease count showed a negative association, with each additional condition associated with a 0.049-point decrease in utility (95% CI: − 0.082 to − 0.017, *p* = 0.01). Positive associations were observed for less remote residence (MMM 4: β = 0.099, 95% CI: 0.005–0.193, *p* = 0.04) and performing recommended physical activity (β = 0.131, 95% CI: 0.013–0.249, *p* = 0.03), compared with MMM 5–7 and no physical activity, respectively. Gender, education, age, and employment status were not significantly associated with HRQoL in the multivariable model.

**Table 3:** Factors associated with EQ-5D-5L utility score (*n* = 75)

Variable	Multivariable linear regression (primary analysis)		
	Coefficient	95% CI	*P* value
**Age**			
<65 years (ref)			
≥65 years	0.103	[− 0.013, 0.219]	0.08
**Gender**			
Male (ref)			
Female	0.038	[− 0.080, 0.156]	0.53
**Education**			
Secondary or below (ref)			
Post-secondary	− 0.104	[− 0.248, 0.041]	0.16
**Geographic rurality**			
MMM 4	0.099	[0.005, 0.193]	**0.04**
MMM 5,6,7 (ref)			
**Employment status**			
Unemployed/not in labour force (ref)			
Employed	0.119	[− 0.006, 0.244]	0.06
**Level of Physical activity**			
No physical activity (ref)			
Recommended physical activity	0.131	[0.013, 0.249]	**0.03**
**Chronic disease burden**			
Number of chronic diseases	− 0.049	[− 0.082, − 0.017]	**0.01**

(1) CI: Confidence interval; ref: reference category; (2) Linear regression with robust standard errors; (3) Bold *p*-values indicate statistical significance (*p* < 0.05)

Table 4 revealed age-stratified patterns in HRQoL and chronic disease burden. Mean HRQoL utility scores demonstrated a U-shaped pattern: 18–54 years (0.843, SD = 0.194), 55–64 years (0.701, SD = 0.287), 65–74 years (0.745, SD = 0.265), and 75 years and above (0.872, SD = 0.119), though overall differences were not statistically significant (*p* = 0.14). This ANOVA test addresses whether age groups differ from each other, not whether each group differs from population norms; the latter comparison is presented in Table 5 using one-sample t-tests against Queensland and Australian population benchmarks. The 55–64 age group demonstrated both the lowest mean HRQoL and highest variability (coefficient of variation 0.409). Chronic disease burden increased significantly across age groups (*p* = 0.04), with mean conditions rising from 2.74 in those aged 18–54 years to 4.14 in those aged 65–74 years. Within age groups, significant negative correlations between HRQoL utility scores and chronic conditions were observed in the 18–54 (*r* = − 0.472, *p* = 0.023), 55–64 (*r* = − 0.489, *p* = 0.018), and 75 year and above (*r* = − 0.820, *p* = 0.012) age groups, though the correlation in the 75 + group should be interpreted with caution given the small sample size (*n* = 8) which limits the reliability of correlation estimates. Age-stratified regressions showed that each additional chronic condition significantly reduced HRQoL in the 18–54 (β = − 0.076, CI: − 0.147, − 0.004, *p* < 0.05) and 55–64 (β = − 0.081, CI: − 0.116, − 0.046, *p* < 0.001) age groups, whilst physical activity demonstrated significant positive effects on HRQoL in the 65–74 (β = 0.281, CI: 0.063, 0.500, *p* < 0.05) and 75+ (β = 0.202, CI: 0.109, 0.295, *p* < 0.01) age groups, though the 75 + age group result is based on only 8 participants with only 2 reporting no physical activity, limiting the reliability of this estimate.

**Table 4:** Age-stratified analysis of characteristics, chronic disease burden, and health-related quality of life

Characteristic	18–54 years (n = 23)	55–64 years (n = 23)	65–74 years (n = 21)	75 years and above (n = 8)	*p*-value^1^
**Health-related quality of life**					
Mean utility score (SD^2^*)*	0.843 (0.194)	0.701 (0.287)	0.745 (0.265)	0.872 (0.119)	0.14
Coefficient of variation	0.231	0.409	0.356	0.137	
Utility range	[0.174 to 1]	[− 0.066 to 1]	[0.087 to 1]	[0.657 to 1]	
**Chronic disease burden**					
Mean number of conditions (SD)	2.74 (1.21)	3.74 (1.74)	4.14 (1.88)	3.50 (1.69)	**0.04**
Condition range	[1–5]	[1–8]	[1–8]	[2–6]	
Multiple conditions (≥ 2)	19 (82.6%)	22 (95.7%)	20 (95.2%)	8 (100%)	0.24
**Correlation**^3^: utility vs. chronic conditions	*r* = − 0.472*	*r* = − 0.489*	*r* = − 0.210 NS	*r* = − 0.820*	
**Regression**: Effect of each additional chronic condition, β (95%CI)^4^	− 0.076* (− 0.147, − 0.004)	− 0.081*** (− 0.116, − 0.046)	− 0.024 NS (− 0.086, 0.038)	− 0.033 NS (− 0.082, 0.016)	
**Modifiable risk factors**					
**Level of physical activity**					
Physical activity - Recommended	21 (91.3%)	17 (73.9%)	12 (57.1%)	6 (75.0%)	0.08
Physical activity - None	2 (8.7%)	6 (26.1%)	9 (42.9%)	2 (25.0%)	
**Regression**: Effect of physical activity on utility, β (95%CI)^5^	NT^6^	β = +0.009 NS (− 0.299, 0.318)	β = +0.281* (0.063, 0.500)	β = +0.202** (0.109, 0.295)	
**Smoking status**					
Never smoker	9 (39.1%)	7 (30.4%)	11 (52.4%)	4 (50.0%)	0.25
Ex-smoker	6 (26.1%)	9 (39.1%)	9 (42.9%)	3 (37.5%)	
Current smoker	8 (34.8%)	7 (30.4%)	1 (4.8%)	1 (12.5%)	
**Sociodemographic factors**					
**Gender**					
Male	3 (13.0%)	7 (30.4%)	5 (23.8%)	3 (37.5%)	0.42
Female	20 (87.0%)	16 (69.6%)	16 (76.2%)	5 (62.5%)	
**Relationship status**					
Married/partnered	4 (17.4%)	10 (43.5%)	10 (47.6%)	3 (37.5%)	**< 0.001**
Separated/divorced/widowed	3 (13.0%)	10 (43.5%)	9 (42.9%)	4 (50.0%)	
Never married	16 (69.6%)	3 (13.0%)	2 (9.5%)	1 (12.5%)	
**Employment status**					
Employed	9 (39.1%)	11 (47.8%)	1 (4.8%)	0 (0%)	**0.01**
Retired	1 (4.3%)	4 (17.4%)	17 (81.0%)	8 (100%)	
Unemployed	7 (30.4%)	4 (17.4%)	0 (0%)	0 (0%)	
Not in the labour force	5 (21.7%)	4 (17.4%)	3 (14.3%)	0 (0%)	
Student	1 (4.3%)	0 (0%)	0 (0%)	0 (0%)	

1. *p < 0.05, ** *p* < 0.01, *** *p* < 0.001; *p*-values from: ANOVA for continuous variables (utility score, chronic disease count); Chi-square tests for categorical variables

2. SD = Standard deviation; NS = Not significant; NT = Not tested

3. Correlation coefficients: Pearson correlation between utility and number of chronic conditions within each age group

4. Effect of each additional chronic condition on utility, adjusted for gender and physical activity. Values shown as β coefficient (95% confidence interval) from age-stratified multivariable linear regression with robust standard errors. Negative values indicate utility decreases with additional conditions

5. Effect of recommended physical activity vs. none on utility, adjusted for gender. Values shown as β coefficient (95% confidence interval) from robust linear regression

6. Statistical testing not performed in 18–54 age group due to insufficient variation (91.3% with recommended activity, *n* = 2 with no activity)

**Table 5:** Comparison of health utility scores with Queensland and Australian population norms by sociodemographic and health characteristics

Characteristic	*n*	Sample mean (SD)	Primary analysis			Sensitivity analysis		
			Queensland Norm	QLD utility decrement	QLD *p*-value	Australian norm	AUS utility decrement	AUS *p*-value
**Overall**	75	0.775 (0.246)	0.916	− 0.141	< 0.001	0.860	− 0.085	0.01
**Age groups**								
18–54 years	23	0.843 (0.194)	0.930	− 0.087	0.04	0.845	− 0.002	0.96
55–64 years	23	0.701 (0.287)	0.898	− 0.197	0.01	0.860	− 0.159	0.01
65–74 years	21	0.745 (0.265)	0.898	− 0.153	0.01	0.880	− 0.135	0.03
75 years and above	8	0.872 (0.119)	0.863	0.009	0.82	0.860	0.012	0.78
**Gender**								
Male	18	0.748 (0.294)	0.924	− 0.176	0.02	0.860	− 0.112	0.13
Female	57	0.784 (0.231)	0.909	− 0.125	< 0.001	0.850	− 0.066	0.03
**Relationship status**								
Married/partnered	27	0.781 (0.246)	0.926	− 0.145	0.01	–	–	–
Separated/divorced/widowed	26	0.689 (0.294)	0.872	− 0.183	0.01	–	–	–
Never married	22	0.870 (0.129)	0.917	− 0.047	0.10	–	–	–
**Employment status**								
Employed	21	0.853 (0.163)	0.941	− 0.088	0.02	–	–	–
Retired	30	0.766 (0.279)	0.890	− 0.124	0.02	–	–	–
Unemployed	11	0.817 (0.203)	0.746	0.071	0.27	–	–	–
Not in the labour force	12	0.604 (0.255)	0.893	− 0.289	0.01		–	–
**Smoking status**								
Never smoker	31	0.793 (0.221)	0.932	− 0.139	0.01	0.890	− 0.097	0.02
Ex-smoker	27	0.779 (0.249)	0.902	− 0.123	0.01	0.840	− 0.061	0.22
Current smoker	17	0.735 (0.291)	0.891	− 0.156	0.04	0.800	− 0.065	0.37
**Physical activity**								
No physical activity	19	0.659(0.248)	0.839	− 0.180	0.01	–	–	–
Recommended activity	56	0.814 (0.235)	0.940	− 0.126	< 0.001	–	–	–

1. Study sample: Indigenous Australians with chronic conditions in rural/remote areas (MMM 4–7), *n* = 75

2. **Population norms**: Queensland norms from Endo et al. (2024) [29]; Australian norms from Redwood et al. (2024) [30]. Both using EQ-5D-5L Australian value set

3. **Age norms**: 18–54 calculated as simple average of four sub-groups from each study (QLD = 0.930; AUS = 0.845). Queensland 75 + averaged from two sub-groups (0.863). Other age groups matched directly

4. Employment: Student (n = 1) excluded; ‘Not in workforce’ compared to Queensland ‘Home duties/carer’ norm

5. **Utility decrement** = Sample mean - Population norm. Negative values indicate worse HRQoL than reference population

6. **Statistical test**: One-sample t-tests comparing sample mean to population norm

7. **“—”** indicates norms not available in Australian norm study (relationship status, employment, physical activity)

8. **Sensitivity analysis**: Queensland norms (primary, geographically matched); Australian norms (sensitivity, national reference)

Table 5 presents participants’ utility scores compared to Queensland population norms across sociodemographic and health characteristics. Overall, participants’ HRQoL utility scores were lower than Queensland population norms (0.775 vs. 0.916, decrement = − 0.141, *p* < 0.001). Age-specific comparisons demonstrated that participants aged 18–54 years, 55–64 years, and 65–74 years had substantially lower utility scores compared to population norms: 18–54 years (decrement= -0.087, *p* = 0.04), 55–64 years (decrement = − 0.197, *p* = 0.01, largest reduction), and 65–74 years (decrement = − 0.153, *p* = 0.01). The 75 + age group showed HRQoL utility scores slightly exceeding Queensland population norms (0.872 vs. 0.863, decrement = 0.009, *p* = 0.82). Sensitivity analysis using Australian norms [30] confirmed robustness pattern. The 55–64 age group showed the largest decrements regardless of reference population (Queensland: − 0.197, *p* = 0.01; Australia: − 0.159, *p* = 0.01), while the 75 years and above age group consistently exceeded norms (Queensland: +0.009, *p* = 0.82; Australia: +0.012, *p* = 0.78).

Table 6 quantifies cumulative health burden as lifetime QALY loss, where one QALY equals one year in perfect health. Using undiscounted estimates with Queensland norms (primary analysis), overall mean QALY loss was 3.14 (SD = 6.26). The 55–64 age group faced greatest burden (4.44 QALYs), followed by 18–54 years (3.68 QALYs) and 65–74 years (2.34 QALYs). The 75 years and above age group showed QALY gain (− 0.10), reflecting HRQoL exceeding population norms. Applying discount rates of 3% and 5% reduced overall estimates to 2.12 and 1.71 QALYs, respectively. Sensitivity analysis using Australian norms yielded lower estimates (overall: 1.69 undiscounted; 1.05 with 5% discount), but age-stratified patterns remained consistent: the 55–64 age group showed highest burden (3.58 undiscounted; 2.12 with 5% discount), the 75 years and above age group showed QALY gain (− 0.13. While reference population choice affects magnitude, the fundamental pattern, greatest burden in the 55–64 age group and resilience in the 75 years and above age group, remained robust across both analyses. Substantial individual heterogeneity in QALY loss was evident across all age groups, with standard deviations often exceeding or approaching mean values (e.g., 18–54 years: mean = 3.68, SD = 8.25; 55–64 years: mean = 4.44, SD = 6.46), reflecting combined variation in utility scores, chronic disease burden, and remaining life expectancy. The broad 18–54 age range uses a midpoint life expectancy estimate (age 35–39: 42.4 years), which masks heterogeneity within this 36-year span. To illustrate this sensitivity: an 18-year-old with the sample mean utility decrement (− 0.087) would experience projected QALY loss of 5.4 years using age-specific life expectancy (61.6 years remaining), compared to 3.7 years using the midpoint estimate, a 45% underestimation. Conversely, a 54-year-old would have QALY loss of 2.5 years using age-specific life expectancy (29.0 years remaining) compared to 3.7 years using the midpoint, a 46% overestimation. These bounds illustrate that individual-level QALY loss estimates within this broad age band should be interpreted as approximations centred on the group mean.

**Table 6:** Lifetime quality-adjusted life year (QALY) loss by age group: Queensland and Australian population norms

Age group	n	Remaining life years	Queensland norms (primary analysis)			Australian norms (sensitivity analysis)		
			Undiscounted	3% Discount	5% Discount	Undiscounted	3% Discount	5% Discount
			Mean (SD)	Mean (SD)	Mean (SD)	Mean (SD)	Mean (SD)	Mean (SD)
18–54 years	23	42.4	3.68 (8.25)	2.07 (4.63)	1.52 (3.40)	0.08 (8.25)	0.04 (4.63)	0.03 (3.40)
55–64 years	23	22.5	4.44 (6.46)	3.19 (4.65)	2.63 (3.82)	3.58 (6.46)	2.58 (4.65)	2.12 (3.82)
65–74 years	21	15.3	2.34 (4.05)	1.86 (3.21)	1.61 (2.78)	2.07 (4.05)	1.64 (3.21)	1.42 (2.78)
75 years and above	8	10.3	− 0.10 (1.23)	− 0.08 (1.05)	− 0.07 (0.94)	− 0.13 (1.23)	− 0.11 (1.05)	− 0.10 (0.94)
Overall	75	25.3	3.14 (6.26)	2.12 (4.07)	1.71 (3.25)	1.69 (6.29)	1.25 (4.11)	1.05 (3.29)

1. **Remaining life years**: Gender-weighted remaining life expectancy using Queensland Indigenous life tables (ABS 2020–2022) [31]. Values shown in the table represent gender-weighted averages calculated using sample gender proportions [18–54 (13.0% male), 55–64 (30.4% male), 65–74 (23.8% male), 75+ (37.5% male)]. Life table assignment approach: 18–54 used midpoint (age 35–39); 55–64 and 65–74 used simple averages of two life table categories [55–64 averaged 55–59 and 60–64; 65–74 averaged 65–69 and 70–74]; 75 + used age 75–79

2. **Calculation method**: Lifetime QALY loss was calculated individually as (Population norm - participant’s EQ-5D-5 L utility score) × remaining life expectancy. Positive values indicate QALY loss (HRQoL below population norm); negative values indicate QALY gain (HRQoL above norm). Undiscounted (0%) estimates assign equal value to all future years of life

## Discussion

This study examined age-stratified HRQoL among Indigenous Australians with chronic conditions enrolled in a virtual primary care service at a rural Indigenous primary healthcare service in Queensland. Three key findings emerged: first, substantial HRQoL disparities exist compared to general population norms across the 18–74 years age group, translating to significant lifetime QALY losses; second, different factors drive HRQoL across the lifespan, with chronic disease burden dominating in adults aged 18–64 years and physical activity may become increasingly important for HRQoL in older age, though this pattern requires confirmation in larger samples; and third, the 75 years and above age group demonstrates exceptional resilience, which may reflect a survivor phenomenon. These findings remained robust across different reference populations and discount rate assumptions, strengthening their validity for informing targeted interventions.

This study found that Indigenous adults aged 18–74 years with chronic conditions demonstrated substantial HRQoL disparities compared to population norms. Prior research has established that multimorbidity substantially reduces HRQoL in Indigenous Australians [8], but limited evidence exists on age-specific patterns and lifetime burden quantification. Our key contributions extend beyond previous work through age-stratified analysis revealing heterogeneous burden distribution, with the 55–64 age group most severely affected, and translation of these disparities into lifetime QALY loss estimates, an approach not previously applied in Indigenous chronic disease research. This provides economic evidence to support age-targeted intervention strategies in Indigenous chronic disease management.

Age-stratified analysis revealed how chronic disease and modifiable behaviours differently affect HRQoL across the lifespan. In younger and middle-aged adults (18–64 years), each additional chronic condition significantly reduced HRQoL, confirming that disease accumulation is associated with HRQoL decline during economically productive years, consistent with evidence indicating that Indigenous Australians experience chronic disease onset approximately 10 years earlier than non-Indigenous Australians [37]. The 55–64 years age group showed the poorest mean HRQoL with nearly half still employed despite carrying the mean chronic disease burden of 3.74 conditions, suggesting this life stage represents peak vulnerability where individuals attempt to maintain workforce participation while managing substantial multimorbidity. In contrast, among older adults (65–74 years and 75 years and above), chronic disease burden no longer predicted HRQoL despite these groups having the most chronic conditions (mean 4.14 and 3.50 respectively), while physical activity emerged as a protective factor in the 65–74 age group (*n* = 21, β = 0.281, *p* < 0.05), though findings should be interpreted cautiously given sample size limitations, particularly in the 75 + group (*n* = 8, with only 2 participants reporting no physical activity). This shift from disease-driven to behaviour-driven determinants suggests age-appropriate intervention strategies: younger adults require chronic disease prevention to delay disease accumulation, while older adults may benefit from physical activity support to maintain function despite existing conditions.

The exceptional HRQoL among participants aged 75 years and above, exceeding both Queensland and Australian population norms despite substantial multimorbidity (mean 3.50 conditions), may reflect a survivor phenomenon: those reaching advanced age with chronic conditions represent an exceptionally robust subset who developed effective disease management strategies and psychological adaptations [38]. Additionally, wellbeing in older Indigenous Australians encompasses dimensions beyond physical health, including cultural connections, family and community involvement, and roles as knowledge keepers [39, 40], which may contribute to maintained quality of life despite chronic disease burden. However, this survivor phenomenon should not diminish concerns about health inequities, it reflects that many Indigenous Australians do not survive to older ages due to earlier disease onset and premature mortality, making early intervention for those aged 18–64 years critically important.

The high proportion of participants reporting recommended physical activity levels (74.7%) warrants discussion given the sample’s chronic disease burden and socioeconomic context. This may reflect several factors. First, social desirability bias in self-reported physical activity is well-documented in health surveys, potentially leading to overestimation of activity levels [41, 42]. Second, cultural interpretations of ‘moderate intensity’ activity may differ in this context, with activities of daily living, cultural practices such as hunting, fishing, or gathering, and community engagement potentially classified as moderate-intensity exercise [43, 44]. Third, genuine engagement in physical activity may be facilitated by the VHS program’s holistic health coaching approach. The reliance on self-reported physical activity without objective measurement limits our ability to verify activity levels or distinguish between these explanations.

### Implication for policy and practice

This study quantifies substantial lifetime QALY losses among Indigenous Australians with chronic conditions, with the 55–64 age group experiencing projected losses of 4.44 QALYs (or 2.63 with 5% discount), providing economic justification for targeted interventions. To contextualise the magnitude of these QALY losses, comparison to published estimates from comparable international populations provides perspective. A US study examining adults aged ≥ 65 years reported lifetime QALY losses of 4.7 years with one chronic condition, rising to 7.9 QALYs with two conditions and 10.8 QALYs with three or more conditions [45]. Similarly, a study of Chinese adults aged ≥ 45 years reported individual-level QALY losses of 3.53 years (95% CI 2.53–4.56) for multimorbidity [46]. Our observed QALY loss of 4.44 years in the 55–64 age group is comparable to these international multimorbidity estimates, indicating a substantial health burden. However, direct comparisons should be interpreted cautiously given differences in population characteristics (age, ethnicity, disease profiles), methodologies, and healthcare system contexts. Notably, no published QALY loss estimates specific for Indigenous Australian populations were identified, representing a significant gap in health equity research that limits our ability to benchmark findings against culturally and contextually appropriate reference points.

While these age-stratified means provide important population-level insights, the substantial individual variability in QALY loss estimates (standard deviations often exceeding mean values) has important policy implications. Age-stratified mean QALY losses identify groups with an elevated burden on average, yet there is significant individual variation within each age group. This suggests that age alone is insufficient for targeting interventions; individual assessments of disease burden and HRQoL may better identify those experiencing greatest lifetime burden and allow for more precise resource allocation.

Virtual primary care with remote monitoring offers promising mechanisms for continuous disease surveillance and early detection of deterioration, potentially intercepting disease progression and reducing lifetime QALY loss. However, effectiveness depends on adequate resourcing. Policy makers should consider allocating dedicated funding to expand ACCHO-led virtual health services, including monitoring equipment, telecommunications infrastructure, culturally appropriate technology support, and adequate clinical staffing.

Age-stratified findings suggest tailored intervention strategies: intensive chronic disease management through remote monitoring for adults aged 18–64 years, and physical activity support programmes for those aged 65–74 years where physical activity was associated with higher HRQoL. Development of Indigenous-specific EQ-5D-5L population norms would strengthen future evaluations by providing culturally appropriate benchmarks reflecting Indigenous conceptualisations of health.

### Strengths and limitations

This study makes several important contributions to Indigenous health research. First, it addresses a significant evidence gap by examining age-stratified HRQoL patterns among Indigenous Australians with chronic conditions accessing virtual primary care, a population and service delivery model not previously studied. Second, the calculation of lifetime QALY losses provides policy-relevant estimates of economic burden and enables comparison with other health conditions and populations, supporting evidence-based resource allocation decisions. Finally, community-based recruitment through an established Indigenous health service facilitating culturally appropriate engagement, encouraged enhanced participation among a population often under-represented in health research.

Several limitations should be considered. The cross-sectional design limits our ability to establish causal relationships between chronic disease burden, physical activity, and HRQoL changes over time. The 49% response rate may introduce non-response bias. We were unable to compare responders with non-responders, as ethics protocols precluded accessing health service records for non-consenting individuals. If more health-conscious individuals participated, our estimates could overstate population HRQoL, while underestimate QALY losses, limiting claims of representativeness to the broader Goondir VHS population. Sample size constraints, particularly in the 75 years and above age group (*n* = 8), limit the reliability of estimates for this cohort. The regression results for this group, while statistically significant, are driven by few observations (including only 2 participants reporting no physical activity) and should be considered hypothesis-generating rather than definitive. The tight confidence intervals likely reflect the specific sample distribution rather than precise population estimates. Similarly, the strong correlation between chronic disease count and utility in the 75 + age group (*r* = − 0.820, *p* = 0.012) has limited statistical reliability with only 8 observations. The limited sample sizes in age-stratified regression analyses mean findings about age-varying determinants should be interpreted cautiously. The broad 18–54 age category may mask heterogeneity across this 36-year span, though sample size precluded finer stratification. The midpoint life expectancy approximation for this group underestimates QALY loss for younger participants (by up to 45% at age 18) and overestimates for older participants (by up to 46% at age 54), though the group-level mean remains a reasonable summary estimate. Self-reported chronic conditions and physical activity are subject to measurement error and recall bias. Furthermore, physical activity was assessed via self-report without objective verification, which may be subject to social desirability bias or differing cultural interpretations of moderate-intensity activity. The notably high proportion reporting recommended activity levels may reflect these measurement limitations. An important consideration is the use of general population norms as comparators, as both Queensland and Australian reference populations are predominantly non-Indigenous and may reflect different health experiences than Indigenous Australians. The eight-year life expectancy gap and earlier onset of chronic diseases suggest that what constitutes “normal” health may differ between populations. Additionally, EQ-5D-5L was developed within a Western biomedical framework and may not fully capture dimensions of wellbeing important to Indigenous peoples, such as connection to Country, cultural identity, and community roles. While sensitivity analysis demonstrated consistent patterns across reference populations, the absence of Indigenous-specific population norms means disparity estimates should be interpreted cautiously. Development of such norms would provide more appropriate benchmarks for future research and strengthen evaluation of health interventions in Indigenous populations.

## Conclusion

Indigenous Australians with chronic conditions accessing rural virtual primary care demonstrate substantial health burden, with age-stratified patterns revealing chronic disease accumulation as a predictor of HRQoL in adults aged 18–64 years while physical activity may be increasingly important in those aged 65 years and over. The 55–64 age group faces greatest burden, experiencing poor HRQoL while maintaining workforce participation with substantial multimorbidity. In contrast, the 75 years and above age group appears to demonstrate resilience, consistent with a survivor phenomenon. Virtual care delivery models may benefit from incorporating age-appropriate intervention strategies addressing this continuum of health challenges while recognising that standard population norms may inadequately reflect Indigenous health contexts. Development of Indigenous-specific population norms would provide a stronger foundation for future health equity research. While our cross-sectional design precludes causal inference, these associations suggest that chronic disease burden and physical activity represent important targets for early intervention in adults aged 18–64 years to address chronic disease accumulation and reduce associated lifetime QALY losses, with the potential to ultimately contribute to closing the health gap between Indigenous and non-Indigenous Australians.

## Data Availability

The data that support the findings of this study are available from the corresponding author upon reasonable request.
